# Establishment of novel specific assay for short‐form glucose‐dependent insulinotropic polypeptide and evaluation of its secretion in nondiabetic subjects

**DOI:** 10.14814/phy2.14469

**Published:** 2020-05-29

**Authors:** Yasutaka Takeda, Yukihiro Fujita, Tsuyoshi Yanagimachi, Nobuhiro Maruyama, Ryoichi Bessho, Hidemitsu Sakagami, Jun Honjo, Hiroki Yokoyama, Masakazu Haneda

**Affiliations:** ^1^ Division of Metabolism and Biosystemic Science Department of Internal Medicine Asahikawa Medical University Asahikawa Japan; ^2^ Division of Diabetology, Endocrinology and Nephrology Department of Internal Medicine Shiga University of Medical Science Otsu Japan; ^3^ Immuno‐Biological Laboratories Co., Ltd. Fujioka Japan; ^4^ Jiyugaoka Medical Clinic, Internal Medicine Obihiro Japan

**Keywords:** cookie meal test, DPP‐4 inhibitor, ELISA, GIP (1–30), oral glucose tolerance test

## Abstract

The short‐form glucose‐dependent insulinotropic polypeptide (GIP) (1–30) is released from islet alpha cells and promotes insulin secretion in a paracrine manner in vitro. However, it is not well elucidated how GIP (1–30) is involved in glucose metabolism in vivo, since a specific assay system for GIP (1–30) has not yet been established. We first developed a sandwich enzyme‐linked immunosorbent assay (ELISA) specific for GIP (1–30) by combining a novel antibody specific to the GIP (1–30) C terminus with the common antibody against GIP N terminus. Then, we explored cross‐reactivities with incretins and glucagon‐related peptides in this ELISA. GIP (1–30) amide, but not GIP (1–42), GLP‐1, or glucagon increased absorbance in a dose‐dependent manner. We next measured plasma GIP (1–30) concentrations in nondiabetic participants (ND) during a 75‐g oral glucose tolerance test or cookie meal test (carbohydrates 75 g, lipids 28.5 g, proteins 8.5 g). Both glucose and cookie load increased GIP (1–30) concentrations in ND, but the increases were much lower than those of GIP (1–42). Furthermore, the DPP‐4 inhibitor significantly increased GIP (1–30) concentrations similarly to GIP (1–42) in ND. In conclusion, we for the first time developed an ELISA specific for GIP (1–30) and revealed its secretion in ND.

## Graphical abstract

For the first time, we established a novel ELISA specific for GIP (1–30). GIP (1–30) secretion is promoted by oral glucose and mix meal load in nondiabetics. DPP‐4 inhibitors increased peripheral blood GIP (1–30) levels.

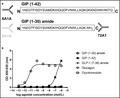

## 1 INTRODUCTION

Glucose‐dependent insulinotropic polypeptide (GIP) was purified from porcine gut mucosal extracts and was originally termed “gastric inhibitory polypeptide” based on its gastric acid inhibitory activity (Brown, Mutt, & Pederson, 1970; Brown, Pederson, Jorpes, & Mutt, 1969). In the 1970s, GIP was shown to be released by oral glucose (Cataland, Crockett, Brown, & Mazzaferri, 1974) and then demonstrated to enhance insulin secretion during intravenous glucose challenge in humans (Dupré, Ross, Watson, & Brown, 1973). Presently, GIP is established as an incretin, which consists of 42 amino acids, and is secreted from intestinal K cells upon food intake and promotes insulin secretion from pancreatic beta cells in a glucose‐dependent manner (Baggio & Drucker, 2007). In addition to its insulinotropic action to beta cells, GIP has been reported to have various physiological effects such as suppression of bone resorption (Nissen et al., 2014), stimulation of fat deposition (Asmar et al., 2010), and amplification of glucagon secretion during hypoglycemia (Christensen et al., 2015; Christensen, Vedtofte, Holst, Vilsbøll, & Knop, 2011).

Ugleholdt et al. showed that prohormone convertase (PC) 1/3, but not PC2, was essential for proGIP to GIP processing in intestinal K cells (Ugleholdt et al., 2006). They also indicated that PC2 could cleave proGIP to “other GIP fragments,” which were not present in intestinal extracts, using PC2‐transfected GH4 cells and pancreatic alpha cell line α‐TC1.9 cells (Ugleholdt et al., 2006).

Several initial reports showed that GIP immunoreactivity was observed in the islet alpha cells (Ahrén, Håkanson, Lundquist, & Sjölund, 1981; Alumets, Håkanson, O'Dorisio, Sjölund, & Sundler, 1978; Smith, Merchant, Johnson, Fujimoto, & Williams, 1977). Afterward, those observations were suspected due to the homology between GIP and glucagon and then GIP was concluded not to be a constituent of the mammalian pancreas, after development of monoclonal antibody of the C‐terminus of GIP (1–42) (Buchan, Ingman‐Baker, Levy, & Brown, 1982). Fujita and colleagues reported that “short‐form” GIP—GIP (1–30), which consists of 30 amino acids—is localized in the islet alpha cells and promotes insulin secretion in a paracrine manner (Fujita, Wideman, et al., 2010). They also showed that GIP (1–30) retained insulinotropic activity equivalently to GIP (1–42) by using perfused mouse pancreas (Fujita, Wideman, et al., 2010). Immunoreactive and bioactive GIP was actually detected from the isolated pancreatic islets, whose release was enhanced by arginine (Fujita, Wideman, et al., 2010). Additionally, we previously showed that GIP (1–30) expression in the islet was enhanced concomitantly with the alpha cell expansion in several rodent models of diabetes and exogenous PEGylated GIP (1–30) injection ameliorated hyperglycemia without weight gain via alleviation of both beta cell death and alpha cell expansion in the low‐dose streptozotocin‐treated diabetic mice (Yanagimachi, Fujita, Takeda, Honjo, Atageldiyeva, et al., 2016).

We assume that GIP (1–30) could play important roles in glucose metabolism. However, the research for GIP (1–30) in humans is still limited, since specific assay systems for GIP (1–30) have not been established. Thus, here, we first developed a sandwich enzyme‐linked immunosorbent assay (ELISA) system specific for GIP (1–30), evaluated its specificity and then measured plasma GIP (1–30) concentrations in nondiabetic participants.

## 2 MATERIALS AND METHODS

### 2.1 Preparation of antibody to human GIP (1–30) amide

First, we established novel rat monoclonal antibodies specifically to C terminus of GIP (1–30) amide. Six‐week‐old female Wister rats were injected subcutaneously with 50 µg of synthetic GIP (24–30) amide peptides CNWLLAQK‐NH2, which were coupled with bovine thyroglobulin as a carrier protein in complete Freund's adjuvant. Three additional injections of immunogen in incomplete Freund's adjuvant were followed every other week. Ten days after final injection, rats were boosted with 20 µg of immunogen and lymphocytes from the immunized rats were fused with myeloma cell line X63‐Ag8.653. Hybridoma cells were selected in hypoxanthine/aminopterin/thymidine medium. Reactive clones against GIP (1–30) amide were selected by sandwich ELISA using a rabbit polyclonal antibody against synthetic peptide corresponds to GIP (3‐17) which was prepared in IBL. Several positive clones were selected by the limited dilution method and 72A1 were finally selected from among the selected clones as chosen for ELISA.

### 2.2 Establishment of the ELISA system for quantification of human GIP (1–30) amide

**FIGURE 1:**
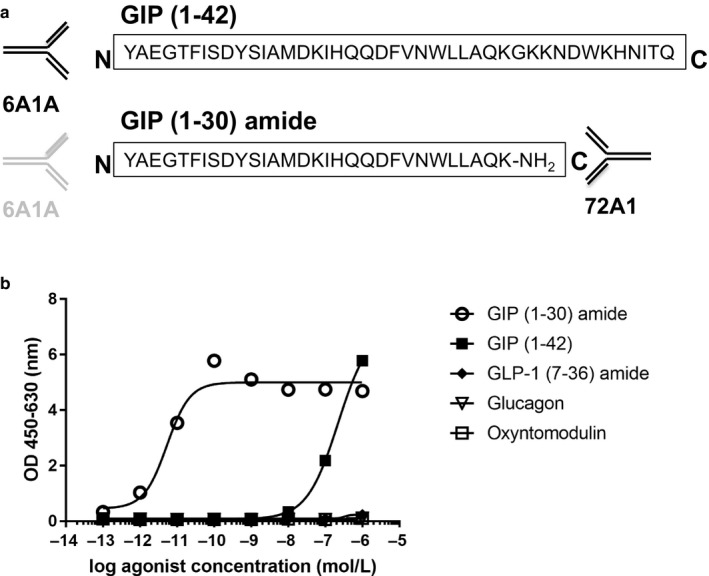
Establishment of the sandwich ELISA system specific for GIP (1–30). (a) Schematic diagram of antibody preparation. 6A1A is an antibody that binds to the N terminus of GIP (1–42) that is already available. 72A1 is a novel antibody to the GIP (1–30) C terminus. (b) Cross‐reactivities among incretins and glucagon‐related peptides in the ELISA system. We examined three independent experiments in assessment of cross‐reactivities among incretins and glucagon‐related peptides. The data are presented as means ± *SEM*. OD, optical density

We developed a sandwich ELISA for GIP (1–30) amide, which consisted of two antibodies, 6A1A and 72A1 (Figure 1a). 6A1A is a mouse monoclonal antibody that binds to the N terminus of active forms of GIP such as GIP (1–42) and GIP (1–30) (27201, IBL). 72A1 was used as a capture antibody specifically to C terminus of GIP (1–30) and HRP conjugated 6A1A Fab’ was used as a detection antibody. Microtiter plates (96 wells) were coated by being filled with 100 μl/well of 50 mM Tris‐HCl buffer (pH 7.5) containing 0.1 µg/well of streptavidin overnight at 4°C. Next, the plates were washed with PBS and blocked with 200 μl/well of 1% (w/v) bovine serum albumin (BSA) in PBS containing 0.05% NaN_3_ overnight at 4°C. After washing with PBST two times, biotinylated 72A1 was reacted for 1 hr at 25°C. Then, the test samples and synthetic peptide of human GIP (1–30) amide as a standard serially diluted with dilution buffer per 100 μl were added into the wells of the coated microtiter plates in duplicate and were incubated at 4°C overnight. After washing with PBST four times, 100 μl of HRP conjugated 6A1A mouse IgG Fab’ was added to each well and incubated at 4°C for 60 min. The wells were washed with PBST five times, and then 100 μl of freshly prepared tetramethyl benzidine solution was added to each well as a substrate. After this process, the plates were incubated in the dark for 30 min at room temperature. The reaction was terminated by adding of 100 μl of 1N H_2_SO_4_. Absorbance of the solution at 450 nm and 630 nm was measured using a microplate reader (Multiskan Go, Thermo Fisher Scientific). The concentration of unknown samples was calculated from standards using the recombinant human GIP (1–30) amide. We also examined the cross‐reactivities with incretins and glucagon‐related peptides in the ELISA system, using the synthetic peptides of GIP (1–42), GLP‐1 (7–36) amide, glucagon and oxyntomodulin (each in the range 10^–13^ to 10^–6^ mol/L).

### 2.3 Study protocol for GIP (1–30) measurement in nondiabetic participants

We recruited five nondiabetic participants (male/female, 5/0; age, 33.4 ± 1.9 years; BMI, 23.2 ± 0.6 kg/m^2^; HbA1c 5.7 ± 0.1%) for a 75‐g oral glucose tolerance test (OGTT) and eight nondiabetic participants (male/female, 2/6; age, 64.4 ± 5.0 years; BMI, 26.6 ± 1.6 kg/m^2^; HbA1c 5.7 ± 0.1%) for a cookie meal test (CMT) with written informed consent. All procedures were performed in accordance with the Declaration of Helsinki, and the protocol of this study was approved by the ethics committee of Asahikawa Medical University (Approval number: 18,202).

OGTT was performed after 10–12 hr overnight fast. Blood samples were collected at 0, 15, 30, 60, 90, and 120 min for plasma glucose measurements and plasma insulin and glucagon determinations. We also collected blood samples at 0, 30, 60, 90, and 120 min for GIP (1–30) and incretins determinations. We further conducted a second OGTT under dipeptidyl peptidase‐4 (DPP‐4) inhibitor treatment (on one day after single administration of 25 mg Omarigliptin (MSD, Tokyo, Japan)). Cookie meal test was also performed after 10–12 hr overnight fast. We employed the cookie meal consisting of 75 g carbohydrate, 28.5 g fat, and 8 g protein for a total of 592 kcal a carton (Meal Test S, Saraya, Osaka, Japan). During CMT, blood samples for plasma glucose, insulin, glucagon, GIP (1–30), and incretins were collected at 0, 30, 60, and 120 min. For plasma GIP (1–30) and incretins determinations, we used special tubes (P800, BD, Tokyo, Japan) at blood collection to avoid inactivation. The plasma samples were separated by centrifugation (3000 RPM for 15 min) at 4°C and stored at −80°C until assays. We evaluated each plasma concentrations and secretions during both OGTT and CMT using the area under the curve (AUC). The AUC was calculated by the trapezoidal method.

### 2.4 Assays

We used the following commercially available ELISA assays: insulin ELISA kit (10‐1113‐01, Mercodia), glucagon ELISA kit (10‐1271‐01, Mercodia), active GIP ELISA kit (27201, IBL), total GIP ELISA kit (EZHGIP‐54K, Millipore), and total GLP‐1 ELISA kit (EZGLP1T‐36K, Millipore), respectively.

### 2.5 Peptides

The synthetic peptides of GIP (1–42), GLP‐1 (7–36) amide, and glucagon were purchased from Peptide Institute (Osaka, Japan). GIP (1–30) amide and oxyntomodulin were purchased from Phoenix Pharmaceuticals (Burlingame, CA, USA).

### 2.6 Statistical analysis

Data are expressed as mean ± *SEM*. Statistical analysis was performed by using paired *t* test between two groups. Data were analyzed using GraphPad Prism 7 (GraphPad Software Inc). A *p*‐value < .05 was considered statistically significant.

## 3 RESULTS

### 3.1 Establishment of the ELISA system specific for GIP (1–30)

First, we examined cross‐reactivities with incretins and glucagon‐related peptides in our ELISA system. Absorbance in the ELISA increased in a dose‐dependent manner by addition of GIP (1–30) amide, but not GIP (1–42), GLP‐1 (7–36) amide, glucagon, or oxyntomodulin (Figure 1b). Accordingly, we established a sandwich ELISA specific for GIP (1–30).

### 3.2 GIP (1–30) secretion during OGTT in nondiabetic participants

**FIGURE 2:**
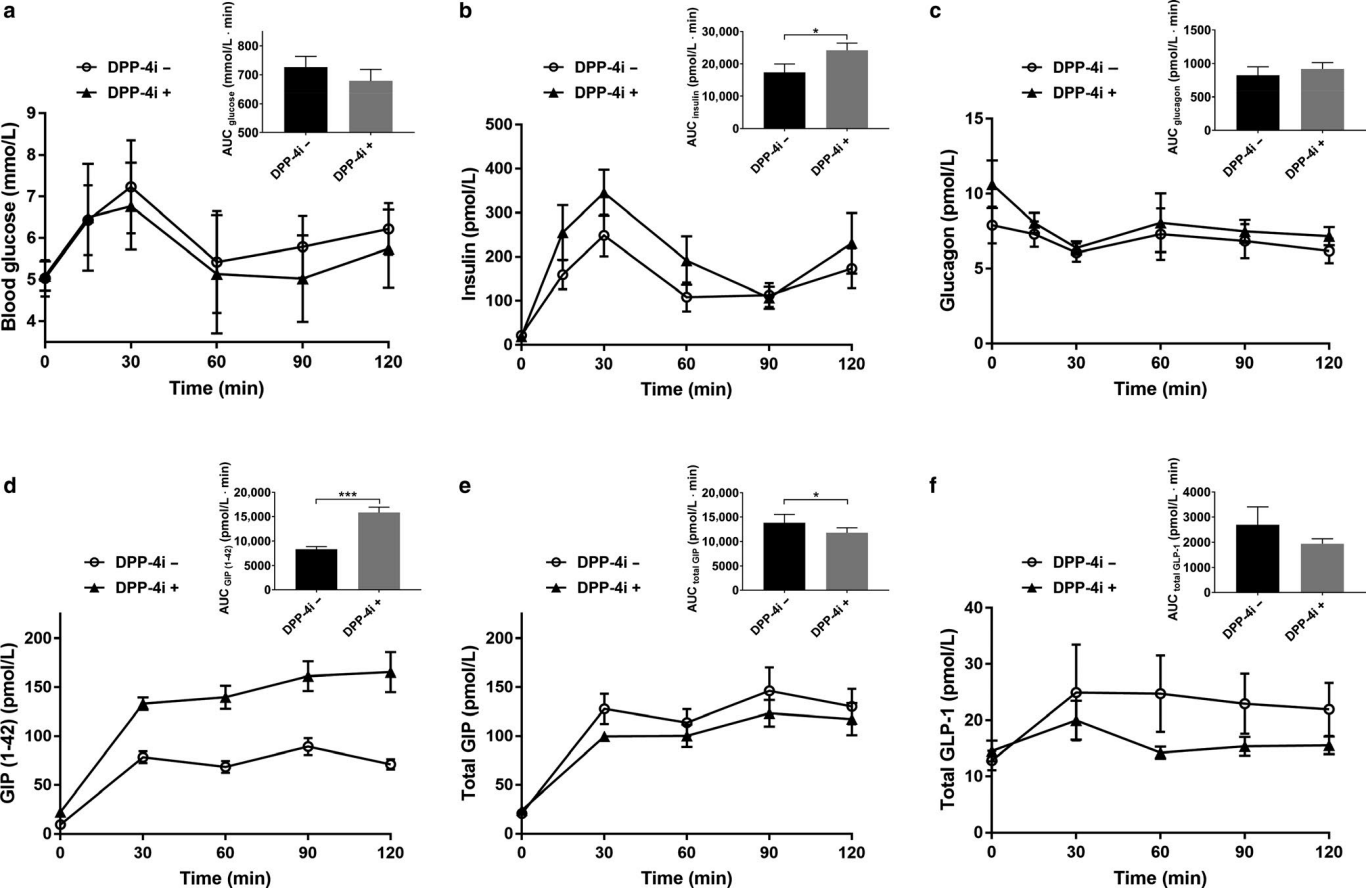
Blood glucose, insulin, glucagon, and incretin levels during OGTT before and after DPP‐4 inhibitor treatment. (a) Blood glucose. (b) Insulin. (c) Glucagon. (d) GIP (1–42). (e) Total GIP. (f) Total GLP‐1. *n* = 5 (male/female, 5/0). The data are presented as means ± *SEM*. AUC, area under the curve. Statistical analysis was performed by using paired *t* test between two groups. *p < .05, ***p < .001 versus before DPP‐4 inhibitor treatment

We performed 75‐g OGTT to evaluate GIP (1–30) secretion in response to oral glucose load and to verify the difference in GIP (1–30) levels with or without administration of DPP‐4 inhibitor. Blood glucose levels peaked at 30 min during OGTT, regardless of DPP‐4 inhibitor treatment in nondiabetic participants. We observed no significant difference in AUC for glucose between before and after treatment with DPP‐4 inhibitor (726.7 ± 36.5 mmol/L·min vs. 678.7 ± 40.1 mmol/L·min, *p* = .2723, Figure 2a). Insulin secretion during OGTT assessed by AUC significantly increased after DPP‐4 inhibitor treatment (17,399 ± 2,589 pmol/L·min vs. 24,169 ± 2,218 pmol/L·min, *p* = .038, Figure 2b). Plasma glucagon levels after oral glucose load decreased both before and after treatment with DPP‐4 inhibitor. No significant difference was observed in AUC for glucagon between before and after treatment with DPP‐4 inhibitor (822.8 ± 127.4 pmol/L·min vs. 918.8 ± 94.9 pmol/L·min, *p* = .1489, Figure 2c).

Plasma GIP (1–42), total GIP, and total GLP‐1 levels increased after oral glucose load. GIP (1–42) secretion assessed by AUC significantly increased after DPP‐4 inhibitor treatment (8,304 ± 561.1 pmol/L·min versus 15,844 ± 1,096 pmol/L·min, *p* = .0006, Figure 2d). In contrast, both AUC for total GIP and AUC for total GLP‐1 decreased after DPP‐4 inhibitor treatment (total GIP, 13,898 ± 1645 pmol/L·min versus 11,816 ± 1,024 pmol/L··min, *p* = .04, Figure 2e; total GLP‐1, 2,701 ± 703.3 pmol/L··min versus 1941 ± 196.9 pmol/L·min, *p* = .2685, Figure 2f), as we previously reported (Yanagimachi, Fujita, Takeda, Honjo, Sakagami, et al., 2016).

**FIGURE 3:**
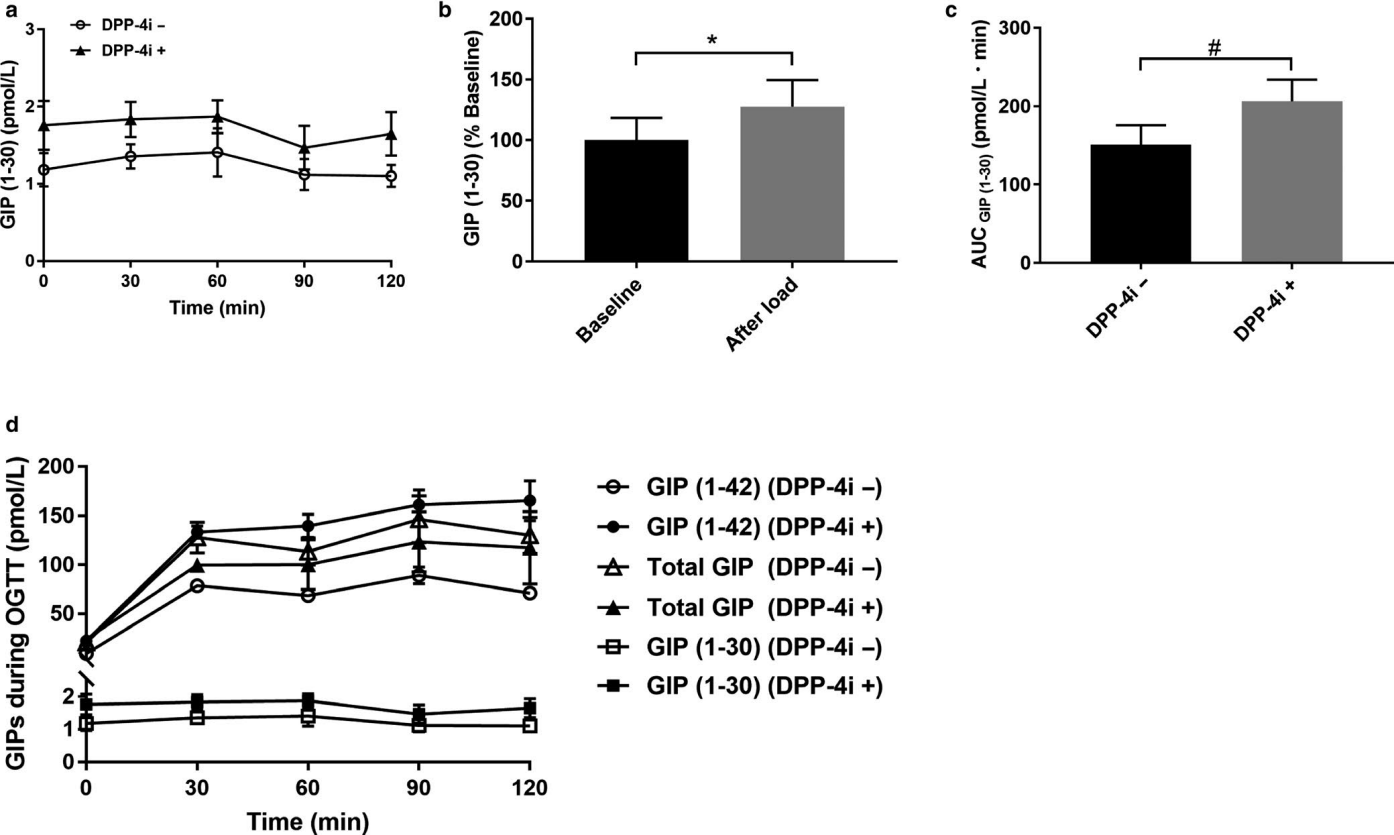
GIP (1–30) secretion during OGTT. (a) GIP (1–30) levels during OGTT before and after DPP‐4 inhibitor treatment. (b) GIP (1–30) secretion before and after oral glucose load without DPP‐4 inhibitor. Baseline, before glucose load; After load, peak value after glucose load. (c) AUC for GIP (1–30) during OGTT with or without DPP‐4 inhibitor. (d) GIP (1–42), total GIP and GIP (1–30) levels during OGTT. *n* = 5 (male/female, 5/0). The data are presented as means ± *SEM*. Statistical analysis was performed by using paired *t* test between two groups. *p < .05 versus baseline. ^#^ *p* < .05 versus before DPP‐4 inhibitor treatment

Then, we evaluated GIP (1–30) secretion by our ELISA system (Figure 3a). Interestingly, plasma GIP (1–30) levels increased about 1.3 times after oral glucose load compared with baseline in nondiabetic participants, indicating that oral glucose ingestion promotes GIP (1–30) secretion in human (baseline vs. peak after load, *p* = .0194, Figure 3b). Furthermore, GIP (1–30) secretion assessed by AUC significantly increased after DPP‐4 inhibitor treatment (150.8 ± 24.8 pmol/L·min vs. 206.4 ± 27.3 pmol/L·min, *p* = .0197, Figure 3c), similarly to GIP (1–42) and GLP‐1. The increases of GIP (1–30) concentrations during OGTT were much lower than those of GIP (1–42) (GIP (1–30) without DPP‐4 inhibitor, baseline, 1.2 ± 0.2 pmol/L, peak after load, 1.5 ± 0.3 pmol/L; GIP (1–42) without DPP‐4 inhibitor, baseline, 9.5 ± 0.5 pmol/L, peak after load, 95.5 ± 9.5 pmol/L, Figure 3d).

### 3.3 GIP (1–30) secretion during CMT in nondiabetic participants

**FIGURE 4:**
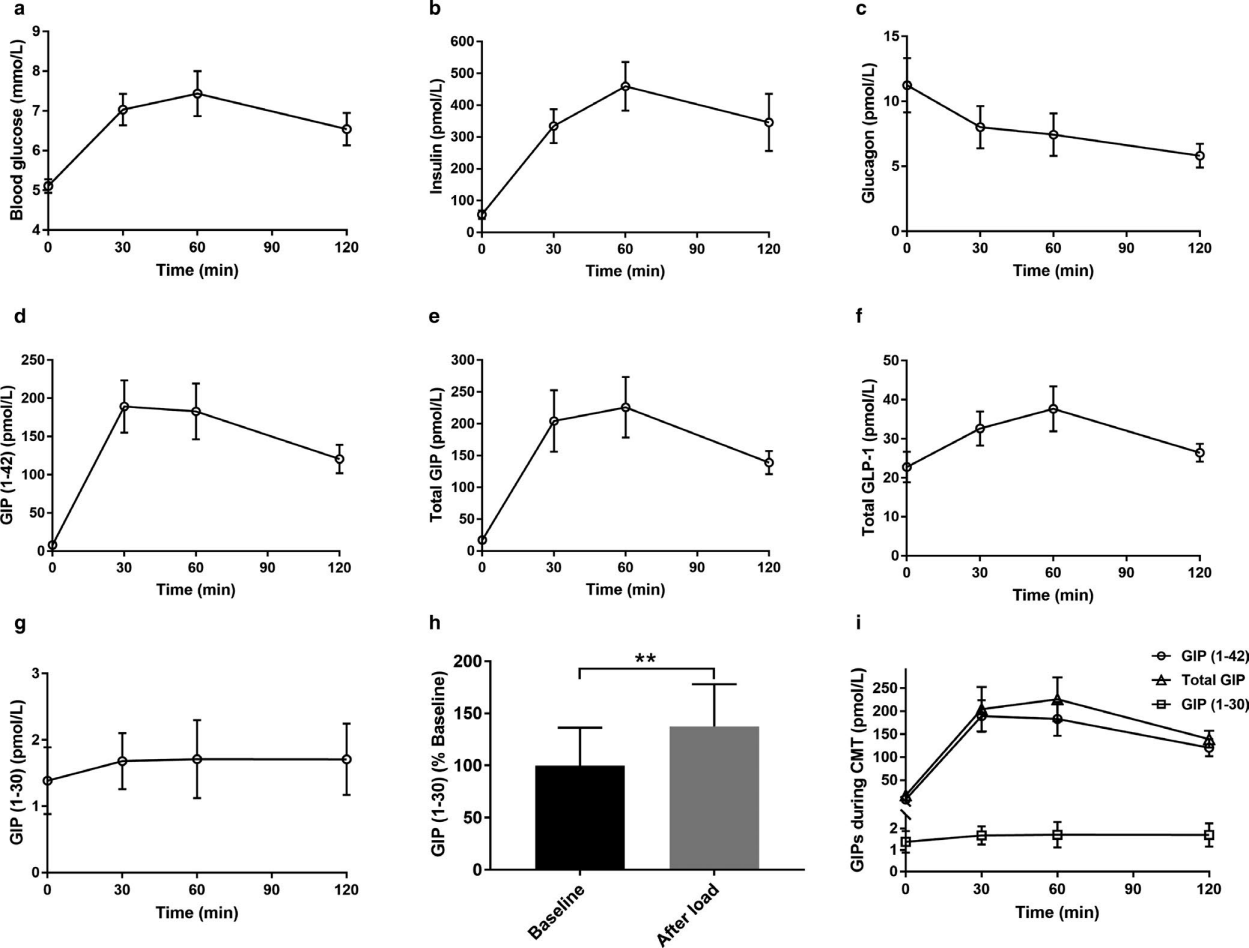
Blood glucose, insulin, glucagon, incretins, and GIP (1–30) levels during CMT. (a) Blood glucose. (b) Insulin. (c) Glucagon. (d) GIP (1–42). (e) Total GIP. (f) Total GLP‐1. (g) GIP (1–30). (h) GIP (1–30) secretion before and after oral cookie load. (i) GIP (1–42), total GIP and GIP (1–30) levels during CMT. *n* = 8 (male/female, 2/6). The data are presented as means ± *SEM*. Statistical analysis was performed by using paired *t* test between two groups. **p < .01 versus baseline

Cookie meal test was also conducted to evaluate GIP (1–30) secretion in response to mixed meal consisted of carbohydrates, lipids, and proteins. Blood glucose, insulin, glucagon, GIP (1–42), total GIP, total GLP‐1, and GIP (1–30) levels during CMT are shown in Figure 4a‐g. Interestingly, we observed that plasma GIP (1–30) levels increased about 1.4 times after oral cookie load compared with baseline (baseline vs. peak after load, *p* = .0078, Figure 4h). Similarly as observed in OGTT study, increases of GIP (1–30) concentrations during CMT were much lower than those of GIP (1–42) (GIP (1–30), baseline, 1.4 ± 0.5 pmol/L, peak after load, 1.9 ± 0.6 pmol/L; GIP (1–42), baseline, 8.2 ± 0.1 pmol/L, peak after load, 204.0 ± 35.2 pmol/L, Figure 4i).

## 4 DISCUSSION

Short‐form GIP (1–30) is released from islet alpha cells and promotes insulin secretion in a paracrine manner in vitro (Fujita, Wideman, et al., 2010). However, the role of GIP (1–30) in glucose metabolism in vivo remains unclear, since a specific assay system for GIP (1–30) has not been established. To the best of our knowledge, this is the first study to develop an ELISA system specific for GIP (1–30) and elucidate GIP (1–30) secretion in human.

First, we developed a sandwich ELISA for GIP (1–30) with our novel antibody to the C terminus of GIP (1–30) amide by combining the N terminus anti‐GIP (1–42). Since absorbance in ELISA increased in a dose‐dependent manner by addition of GIP (1–30) amide but not by GIP (1–42), GLP‐1 (7–36) amide, glucagon, or oxyntomodulin, we consider that our ELISA system is reliable and extremely specific for GIP (1–30).

Next, we conducted OGTT to evaluate GIP (1–30) secretion in response to oral glucose load and to validate the difference in GIP (1–30) secretion with or without DPP‐4 inhibitor. We observed that GIP (1–30) concentration increased after oral glucose load in nondiabetic participants, suggesting that oral glucose ingestion promotes GIP (1–30) secretion in human, similarly to incretins. Moreover, we also observed that GIP (1–30) secretion assessed by AUC increased under DPP‐4 inhibitor treatment. We speculate that DPP‐4 can catalyze N‐terminal 2 amino acids of GIP (1–30), similarly to GIP (1–42), resulting in the higher active GIP (1–30) concentrations. Furthermore, CMT revealed that GIP (1–30) secretion also increased in response to mixed meal load and the secretion was comparable with those during OGTT. This finding indicated that oral ingestion of both glucose and mixed meal were equally important to promote GIP (1–30) secretion in human. Meanwhile, we observed that absolute GIP (1–30) levels and the increments during both OGTT and CMT were much lower than those of GIP (1–42). We speculate that these lower peripheral blood concentrations of GIP (1–30) may likely reflect that GIP (1–30) plays an important role in insulin secretion in a paracrine manner as previously reported (Fujita, Wideman, et al., 2010). Fehmann et al. previously demonstrated that both GIP (1–42) and GIP (1–30) equally stimulate cAMP generation and insulin secretion by using insulin‐secreting beta cell lines. Additionally, they also revealed that both GIP (1–42) and GIP (1–30) equipotently stimulated proinsulin gene expression in beta cell lines (Fehmann & Göke, 1995). Furthermore, Gault and colleagues reported that the same doses of exogenous DPP‐4‐resistant GIP (1–42) and GIP (1–30) equally stimulated insulin secretion and decreased blood glucose levels in mice (Gault, Porter, Irwin, & Flatt, 2011). Based on these reports, we presume the possibility that GIP (1–30) secreted from islet alpha cells contributes to insulin secretion in beta cells as well as GIP (1–42) secreted from small intestine, although peripheral blood concentration of GIP (1–30) is substantially lower.

We consider that GIP (1–30) can be released mostly from the pancreatic alpha cells, since Fujita et al. showed that immunoreactive and bioactive GIP was detected from the isolated pancreatic islets and glucose concentration‐dependent insulin secretion from the isolated islets was suppressed by addition of neutralizing antibody against GIP (1–30) or GIP receptor antibody (Fujita, Wideman, et al., 2010). However, we also need to consider the possibility that GIP (1–30) may be derived from other organs. Lund and colleagues showed that peripheral GIP (1–30) levels during an OGTT in totally pancreatectomized subjects were within the detectable limits of their radioimmunoassay for GIP (1–30) (Lund et al., 2016). Similarly, in our preliminary data, postprandial plasma GIP (1–30) levels in patients with total pancreatectomy were also within the measurable limits of our ELISA (data not shown). These findings suggest that GIP (1–30) may also be secreted from other organs. We speculate the gut endocrine cells as another candidate, since previous study showed that PC2 immunoreactivity and GIP immunoreactivity with the GIP antibody detecting GIP (1–30) were colocalized in the gut endocrine cells differently from the classical K cells with PC1/3 (Fujita, Asadi, Asadi, Yang, Kwok, & Kieffer, 2010).

In the present study, we unveiled for the first time GIP (1–30) secretion in nondiabetic participants. How is GIP (1–30) secretion in subjects with diabetes? We previously reported that GIP (1–30) expression in the islets was enhanced concomitantly with alpha cell expansion in several diabetic mouse models (Yanagimachi, Fujita, Takeda, Honjo, Atageldiyeva, et al., 2016). Hyperglucagonemia in both fasting and postprandial states can contribute to hyperglycemia through increasing hepatic glucose output in type 2 diabetes, which is involved in morphological abnormalities of islet alpha cells (Gromada, Franklin, & Wollheim, 2007). We therefore anticipate that diabetic subjects may show increased GIP (1–30) levels, reflecting alpha cell expansion.

We have limitations in the current study, since our study included only nondiabetic participants and the sample size was small. To clarify pathophysiological roles in glucose metabolism, we are now working to investigate GIP (1–30) secretion in participants with impaired fasting glucose, impaired glucose tolerance, and overt diabetes. We expect to report these new findings about GIP (1–30) soon.

In conclusion, we developed a novel ELISA specific for GIP (1–30) and unveiled that the secretion was promoted by oral glucose and mixed meal load. Furthermore, as is also true of the incretins, GIP (1–30) levels increased upon administration of DPP‐4 inhibitor.

## CONFLICT OF INTEREST

The authors declare that there is no conflict of interest with this manuscript.

## AUTHOR CONTRIBUTIONS

YT, YF, TY, NM, RB, HS, JH, HY, and MH contributed to the study concept and design; YT, YF, TY, NM, JH, and HY acquired data; YT, YF, TY, NM, RB, HS, JH, HY, and MH analyzed and interpreted the data and performed statistical analysis; YT, YF, and NM drafted the manuscript; YT, YF, TY, NM, RB, HS, JH, HY, and MH reviewed the manuscript for important intellectual content.
